# Phonetic Encoding of Coda Voicing Contrast under Different Focus Conditions in L1 vs. L2 English

**DOI:** 10.3389/fpsyg.2016.00624

**Published:** 2016-05-13

**Authors:** Jiyoun Choi, Sahayng Kim, Taehong Cho

**Affiliations:** ^1^ARC Centre of Excellence for the Dynamics of Language, Western Sydney UniversitySydney, NSW, Australia; ^2^Hanyang Phonetics and Psycholinguistics Lab, Department of English Language and Literature, Hanyang UniversitySeoul, South Korea; ^3^Hongik UniversitySeoul, South Korea

**Keywords:** english coda voicing, L2 speech, prominence, focus, prosodic structure, phonetics-prosody interface, Korean learners of English

## Abstract

This study investigated how coda voicing contrast in English would be phonetically encoded in the temporal vs. spectral dimension of the preceding vowel (in vowel duration vs. F1/F2) by Korean L2 speakers of English, and how their L2 phonetic encoding pattern would be compared to that of native English speakers. Crucially, these questions were explored by taking into account the phonetics-prosody interface, testing effects of prominence by comparing target segments in three focus conditions (phonological focus, lexical focus, and no focus). Results showed that Korean speakers utilized the temporal dimension (vowel duration) to encode coda voicing contrast, but failed to use the spectral dimension (F1/F2), reflecting their native language experience—i.e., with a more sparsely populated vowel space in Korean, they are less sensitive to small changes in the spectral dimension, and hence fine-grained spectral cues in English are not readily accessible. Results also showed that along the temporal dimension, both the L1 and L2 speakers hyperarticulated coda voicing contrast under prominence (when phonologically or lexically focused), but hypoarticulated it in the non-prominent condition. This indicates that low-level phonetic realization and high-order information structure interact in a communicatively efficient way, regardless of the speakers’ native language background. The Korean speakers, however, used the temporal phonetic space differently from the way the native speakers did, especially showing less reduction in the no focus condition. This was also attributable to their native language experience—i.e., the Korean speakers’ use of temporal dimension is constrained in a way that is not detrimental to the preservation of coda voicing contrast, given that they failed to add additional cues along the spectral dimension. The results imply that the L2 phonetic system can be more fully illuminated through an investigation of the phonetics-prosody interface in connection with the L2 speakers’ native language experience.

## Introduction

Recent years have witnessed a rapidly growing body of research on the phonetics-prosody interface which illuminates how phonetic realization of segments is fine-tuned by higher-order prosodic structure, and how the prosodic structure is in turn manifested in the fine phonetic detail (e.g., Fougeron and Keating, 1997; de Jong, 2004; Cho and McQueen, 2005; Cho et al., 2014). In a widely received view on the phonetics-prosody interface, high-level prosodic structure is assumed to modulate not only fine-grained phonetic shaping of individual segments, but also phonetic encoding of paradigmatic phonological contrast. A well-known example comes from position-related phonetic modulation whereby a same segment is produced differently as a function of prosodic position (Fougeron and Keating, 1997; Cho and Keating, 2001, 2009). Another example is phonetic modulation due to prosodic structure that involves accent (or prominence in a broader sense). When the prominence of a linguistic unit (such as a syllable or a word) is expressed by a nuclear pitch accent (an element of prosodic structure in English; see Shattuck-Hufnagel and Turk, 1996), phonetic clarity of individual segments is heightened, maximizing phonological contrast through enhancement of phonetic features involved (e.g., de Jong, 1995, 2004; Cho and McQueen, 2005; Cho et al., 2014).

Accumulated evidence on such phonetics-prosody interface has led to a common consensus among researchers that a fuller understanding of the phonetic system of a given language should be accompanied by an understanding of the detailed aspects of the phonetics-prosody interface (see Fletcher, 2010; Cho, 2011, for a review). Theories of the phonetics-prosody interface, however, have been developed primarily based on L1 speech, leaving many relevant questions unanswered in L2 speech. Numerous studies on L2 speech production have indeed vigorously informed how L2 speech production is influenced by L1 phonetic knowledge both on the segmental level (e.g., Flege, 2003; Best and Tyler, 2007) and on the suprasegmental level (e.g., Munro, 1995; Trofimovich and Baker, 2006), but our understanding of the interplay between the two levels in L2 is still at an embryonic stage (cf. Davidson, 2011). In an effort to fill the gap, the present study explores the interplay between low-level phonetic realization and high-order prosodic structure in L1 (English) vs. L2 (by Korean learners of English) by investigating a case of phonetic modulation of coda voicing contrast in English.

The coda voicing contrast in English is known to be phonetically encoded in both the temporal and the spectral dimensions of the preceding vowels as reflected in vowel duration and formants (F1, F2; e.g., Chen, 1970; Wolf, 1978; Keating, 1985; Summers, 1987; Crowther and Mann, 1992; Maddieson, 1997; de Jong, 2004; Moreton, 2004). For example, in the temporal dimension, vowel duration is longer before a voiced than before a voiceless coda; and in the spectral dimension, the vowel (especially the low vowel, /æ/ or /ɑ/) is produced with *lower* F1 (positioning the vowel *higher* in the vowel space), and *higher* F2 (positioning the vowel *more advanced* in the vowel space) before a voiced than a voiceless coda. Given its multi-dimension cues, the case of coda voicing in English allows us to investigate how the cues in different phonetic dimensions are used differentially as a function of L2 speakers’ native language experience.

The goal of the present study is therefore to investigate how the coda voicing contrast in English is indeed manifested in the fine-grained temporal vs. spectral dimensions of the preceding vowel in L1 vs. L2, with a view to understanding the influence of L1 experience on L2 phonetics-prosody interface. For L2 speakers, we chose native Korean (NK) learners of English because Korean differs from English in crucial ways which provide a basis for testing how the non-native coda voicing contrast would be modulated by L2 speakers’ native language experience. Two crucial language-specific aspects relevant for the present study are (1) Korean does not employ voicing contrast in the coda position, and (2) Korean has a much smaller vowel inventory which might reduce their sensitivity to the spectral (formant) cues (see below for further discussion). In what follows, we will develop specific research questions in connection with these language-specific characteristics of Korean along with some predictions that ensue from NK speakers’ native language experience.

The first question to be considered in the present study is how NK L2 speakers of English encode coda voicing contrast in the temporal vs. the spectral dimensions as compared with how NAE speakers do. In Korean, the laryngeal contrast of stops is completely neutralized to a voiceless unreleased stop (see Cho and McQueen, 2006, for a related discussion). This means that NK L2 speakers of English do not have native language experience with coda voicing contrast in any phonetic dimension, whether spectral or temporal. Given the lack of NK speakers’ experience with coda voicing contrast, one might expect that NK speakers would have difficulties in encoding the coda contrast equally in the spectral and in the temporal dimension.

Alternatively, however, NK L2 speakers of English might use the temporal vs. the spectral cues in an asymmetric way. They might rely on the temporal dimension to encode coda voicing contrast, but their use of spectral dimension could be restricted, possibly attributable to the fact that Korean employs a more sparsely populated vowel space compared to English. For example, there are only two contrastive vowels (/i/ and /ε/) in the front region of the vowel space in Korean (e.g., Yang, 1996) as opposed to five in English (/i, ɪ, eɪ, ε, æ/). With the experience of a sparsely populated vowel space in their native language, NK speakers are supposed to be less sensitive to fine-grained changes in the spectral (formant) dimension than NAE speakers are. This possibility is in fact in line with an assumption in the literature that speakers of a language with a sparsely populated vowel space has larger Difference Limens (DLs, or Just Noticeable Differences). For example, native listeners of Japanese (with a relatively smaller vowel inventory) show DLs as large as 13% of the formant frequency (Nakagawa et al., 1982), while DLs as small as 1% has been reported for native listeners of American English (with a larger vowel inventory; Kewley-Port and Watson, 1994; Kent and Read, 2002 for a related discussion; see also Iverson and Evans, 2009, for a study showing an advantage of a complex L1 vowel space in L2 learning). It is therefore plausible to assume that small changes in the spectral dimension are not easily accessible to NK L2 speakers of English, and therefore they do not utilize the spectral cues to the coda voicing contrast, or at least not in as fine-grained a way as the native speakers do.

The cues used in the temporal dimension, on the other hand, appear to be more readily available to L2 speakers as has often been noted by previous researchers (e.g., Flege and Hillenbrand, 1986; Bohn, 1995; Escudero et al., 2009, to name a few). Bohn (1995) proposed that L2 speakers’ propensity to rely more on a temporal cue than on a spectral cue (when both cues are available for an L2 contrast) is attributable to the universally driven perceptual salience of durational cues, although L2 speakers’ native language experience may further modulate the L2 speakers’ use of durational cues (see Broselow and Kang, 2013 for a review; Escudero et al., 2009 for related discussion). For example, Flege and Hillenbrand (1986) showed that native speakers of three languages (French, Swedish, and Finnish) all exploited vowel duration in perceiving the coda voicing contrast between /z/ and /s/ in English, but the effect was smaller for native speakers of French than those of Swedish and Finnish, showing some native language effect. Crucially, speakers of these languages all showed comparable reliance on durational cues to coda voicing, independently of the amount of their exposure to English. This is again consistent with the universally driven perceptual account—i.e., because the durational cues are perceptually salient, the durational cues can be easily exploited by L2 speakers regardless of the speakers’ native language *and* their English proficiency. The salient nature of durational cues may also be reflected in the tendency that listeners rely on durational cues more than F0 cues at prosodic junctures in lexical segmentation when processing an unfamiliar language (e.g., Tyler and Cutler, 2009; Kim et al., 2012). Non-native speakers indeed appear to exploit a temporal cue in L2 even if the specific temporal cue is not directly used in their native language. In Arabic, for example, the stop voicing contrast is maintained in coda position, but Arabic does not systematically use the vowel duration cue to coda voicing presumably because vowel duration is preserved for maintaining phonemic length (quantity) contrast between vowels (see de Jong and Zawaydeh, 2002 for a related discussion). Nevertheless, Arabic L2 speakers of English utilized the vowel duration to encode the coda voicing contrast in English (Flege and Port, 1981).

Taken together, it is reasonable to assume that the phonetic cues are different in nature in terms of whether they are expressed in the temporal vs. the spectral dimensions, so that the former tends to be universally exploitable while the latter is more prone to be language-specifically tuned. Under this view, phonetic cues in the temporal dimension in L2 are more readily accessible to L2 speakers than are those in the spectral dimension, leading to a prediction that NK speakers will be able to encode the coda voicing better in the temporal than in the spectral dimension. Furthermore, given the perceptually driven accessibility of durational cues, one might expect that the NK speakers would show a similar phonetic encoding pattern of coda voicing along the temporal dimension, regardless of their English proficiency. The fact that the coda voicing effect on vowel duration is a near-universal phenomenon (Chen, 1970; Keating, 1985; Maddieson, 1997; Cho, 2015, for a review) indeed appears to reinforce these predictions.

Another important question of the present study concerns how NK L2 speakers of English would express the phonetics-prosody interface as to be reflected in modulation of coda voicing contrast as a function of prominence (which stems from a prosodic structure of a given utterance—e.g., Beckman, 1996; Shattuck-Hufnagel and Turk, 1996; Fletcher, 2010; Cho, 2011). It has been well-documented in the literature that phonological distinction is enhanced or *hyperarticulated* in the prominent (accented) condition while it is attenuated or *hypoarticulated* when the segment occurs in the non-prominent (unaccented) condition (e.g., de Jong, 1995, 2004; Cho and McQueen, 2005; Cho et al., 2011, 2014). For example, de Jong (2004) showed that NAE speakers hyperarticulated (or exaggerated) the durational difference due to coda voicing in order to maximize phonological distinction of the coda voicing contrast (at least in the temporal dimension) in the prominent context, whereas the coda voicing contrast was minimized or hypoarticulated in the non-prominent context. Such an interaction between coda voicing and prominence, however, was not observed in the spectral dimension (F1 and F2), although the coda voicing contrast itself was still reflected in the spectral dimension. In the present study, we extend this study to L2 speech by investigating the extent to which NK L2 speakers of English exploit the acoustic-phonetic space for phonetic encoding of coda voicing contrast as a function of the prominence system of prosodic structure.

If NK L2 speakers of English indeed fail to use the spectral dimension to encode coda voicing contrast, and if the L2 temporal cues are readily accessible to NK speakers, NK speakers are also likely to use the temporal dimension for phonetic modulation of coda voicing contrast as a function of prominence. More crucial questions, however, are how efficiently NK speakers use the temporal dimension along a hypo- to hyper-articulation (H&H) continuum (cf. Lindblom, 1990) to express *both* the phonological voicing contrast and its interaction with the prominence system, and how much the NK speakers’ way of using the temporal dimension is attributable to their native language experience. In order to address these questions in connection with communicative efficiency in L2 (to be reflected in the way that the H&H continuum is exploited by L2 speakers), we integrated the prominence factor with information structure which is often assumed to be mediated by the prominence system of prosodic structure (e.g., de Jong, 2004). That is, the prominence conditions (accented vs. unaccented) were obtained with three different focus types that were assume to stem from information structure, so that we could examine how phonetic encoding of coda voicing could be fine-tuned as a function of information associated with different focus types. Thus, the target-bearing words were produced with one of the following focus types: (1) phonologically contrastive focus in which the coda voicing contrast (*bed* vs. *bet*) was directly emphasized; (2) lexically contrastive focus in which a target-bearing word was contrastive with a semantically related word (*bed* vs. *chair*); and (3) no focus in which the target-bearing word was in the background with a contrastive focus being placed elsewhere in the utterance.

As for the focus effects in L1, de Jong (2004) already showed that different focus types induced different degrees in the coda voicing effect on the preceding vowel duration. The vowel length difference was found to be enhanced when the targets were focused (phonologically or lexically) compared to when the targets received no focus, and more importantly, the focus effect was found to be more robust in the phonological than in the lexical focus condition. This suggests that the phonetics-prosody interface as reflected in enhancement of coda voicing under prominence is further modulated by higher-order information structure, which may be taken to be driven by an optimization of communicative efficiency in response to information structure. That is, it appears that speakers make an articulatory effort focusing on either a particular phonological contrast or the whole lexical item to enhance the locus of information as signaled by information structure (driven by the principle of contrast maximization), while they ease articulation when the target is not the locus of information (driven by the principle of effort minimization; cf. Lindblom, 1990; Flemming, 1995). The present study builds on this assumption in L1, and further explores the extent to which such communicative efficiency may be reflected in L2 production by NK speakers. The L2 system is in fact considered to operate through the interaction between principles of contrast maximization and effort minimization (see Hawkins, 2014, for a related discussion), and one might therefore expect that NK speakers would show an interaction between coda voicing and focus in a way similar to that of NAE speakers, as far as the common goal is concerned—i.e., to achieve communicative efficiency in response to information structure. But as non-native speakers, NK speakers might not be able to show as efficient a pattern as native speakers do, not only because they have less experience with the L2 communicative system as a whole, but also because their production is likely to be affected by their native language experience. As briefly mentioned above, while native (NAE) speakers use both the temporal and the spectral dimension to encode the coda voicing contrast, NK L2 speakers of English are likely to rely exclusively on the temporal dimension to maintain the coda voicing contrast in a communicatively efficient way as regulated by information structure. If this is the case, with the lack of spectral cues to coda voicing contrast, NK speakers’ use of temporal dimension would be restricted to the extent that the phonological voicing contrast in the temporal dimension is not blurred when the system prefers hypoarticulation.

Finally, the present study examines the coda voicing effect on syllable-onset Voice Onset Time (VOT). One of the traditional explanations for the coda voicing effect on the preceding vowel duration may be that the rate of (V-to-C) closure formation for the voiced stop is slower (Chen, 1970), which implies that the temporal effect is localized to a later part of the vowel which roughly corresponds to the closing gesture for the coda. Most recently, however, in an acoustic study, Pycha and Dahan (2016) showed that coda voicing influences the relative timing of the nucleus and the offglide for a diphthong /ɑI/, implying that the effect is not local but global, regulating the temporal organization of the first and the second components of the vowel. The hypothesized global articulatory effect is further consistent with a perceptual account—i.e., an acoustically defined vowel would be lengthened, enhancing the percept of voicing for a voiced coda (see Raphael, 2005, for a review). If the vowel lengthening due to coda voicing is entirely perceptually driven, the lengthening effect does not need to be localized to a later part of the vowel. None of these explanations, however, predicts the coda voicing effect on the syllable-onset VOT, as VOT is not involved in closure formation of the coda, nor does it contribute to the voiced percept or the nucleus-offglide timing as it is by nature voiceless. From an articulatory point of view, however, the onset of the vocalic (mouth opening) gesture for the vowel (i.e., the release of closure) coincides with the onset of VOT and therefore VOT may be considered as a ‘voiceless’ part of the vowel (for example, in the framework of Articulatory Phonology, Browman and Goldstein, 1992). If the coda voicing effect on the vowel is localized near the coda consonant, it is expected to influence the closing gesture for the coda, but not the vowel’s opening gesture that includes VOT in the vowel’s temporal domain. In such a case, VOT will not vary as a function of coda voicing. Alternatively, if the coda voicing effect influences the temporal structure of the entire vowel including the vowel’s opening gesture, VOT as part of the vowel is expected to be longer before a voiced coda just like the acoustic vowel duration is. In the present study, we test this possibility in both L1 and L2 speech.

## Materials and Methods

### Participants and Recording

Thirty-six speakers participated in the study for monetary reward. They were 12 native speakers of American English (six females, six males, aged: 21–33, mean age = 26), 12 Korean advanced learners of English with an average TOEFL score of 110 (six females, six males, aged: 21–26, mean age = 23), and 12 Korean intermediate learners of English with an average TOEFL score of 75 (six females, six males, aged: 21–28, mean age = 24). The native speakers of English were exchange students, English teachers or visitors residing in Seoul at the time of recording. The Korean learners of English were all university students. All participants were naïve as to the purpose of the present study. The speech data were recorded in a soundproof booth at the Hanyang Phonetics and Psycholinguistics Lab, with a Tascam HP-Ps digital recorder and a SHURE KSN44 microphone at a sampling rate of 44.1 kHz.

### Speech Materials and Procedure

Four minimal pairs of English CVC words differing in the voicing of coda stops were used as in (1):

(1) (a) front mid vowel /ε/: *bed-bet, ped-pet*
      (b) front low vowel /æ/: *bad-bat, pad-pat*

Each of the eight target words (in the four pairs) in two different vowel contexts (/ε, æ/) was placed in a carrier sentence, which was an answer to a question in a mini discourse situation. The mini discourse was used to induce the desired variety of accent-placement patterns with different focus types and prosodic groupings. Example sentences with a target word *bed* are given in **Table 1**.

**Table 1:** Example sentences with a target *bed*.

IP-initial (IPi)	PH-FOC	A: *Did you write ‘BET fast again’?*
		B: *Not exactly. ‘BED fast again’ was what I wrote.*
	LEX-FOC	A: *Did you write ‘CHAIR fast again’?*
		B: *Not exactly. ‘BED fast again’ was what I wrote.*
	NoFOC	A: *Did you write ‘bed SLOWLY again’?*
		B: *Not exactly. ‘bed FAST again’ was what I wrote.*
IP-medial (IPm)	PH-FOC	A: *Did you write ‘say BET fast again’?*
		B: *No, I wrote ‘say BED fast again.’*
	LEX-FOC	A: *Did you write ‘say CHAIR fast again’?*
		B: *No, I wrote ‘say BED fast again.’*
	NoFOC	A: *Did you write ‘say bed SLOWLY again’?*
		B: *No, I wrote ‘say bed FAST again.’*

*The target word is underlined. Focused words are in uppercase letters*.

As can be seen in the table (underlined) target words always occurred in the second sentence (‘B’) preceded by a prompt question (‘A’) which was used to induce an intended focus type for the target word. Following de Jong and Zawaydeh (2002) and de Jong (2004), the focus types were manipulated as follows (see Gussenhoven, 2007 for a comprehensive review of focus types):

– ***phonological (segmental) focus*** (PH-FOC): with a corrective contrastive focus on the phonological voicing of codas between the target word and the contrastive counterpart in the prompt question which form a minimal pair (e.g., *BET* vs. *BED*).
– ***lexical focus*** (LEX-FOC): with a corrective contrastive focus on the target word which is semantically related to the contrasting word in the question (e.g., *CHAIR* vs. *BED*).
– ***no focus*** *(background;* No FOC): with a corrective contrastive focus on a word following the target word, so that the target word became the background in the focus-background structure (e.g., *bed SLOWLY* vs. *bed FAST*).

As can be seen in **Table 1**, the position of the target word was controlled to be either in the initial or in the medial position of the Intonational Phrase (IP), because prosodic position may interact with prominence. The target word was placed either in the initial position of a sentence (e.g., *Not exactly. ‘****Bed*** *fast again’ was what I wrote*), which is likely to be the beginning of an IP, or in the middle of an IP (e.g., *No. I wrote ‘say* ***bed*** *fast again’*), given the likelihood that the phrase *‘say bed fast again’* forms an IP.

The prompt questions were pre-recorded by a female native speaker of American English who had been trained to produce intended focus-inducing patterns. During the recording, subjects first silently read the question-and-answer sentences on a computer screen. They then heard the pre-recorded prompt question, and answered it aloud as written on the screen. The first 36 trials were practice trials, so that speakers familiarized themselves with focus types in different mini discourse situations. They were asked to speak casually at a comfortable speech rate as if they were talking to a friend. The practice session was repeated when a speaker was not fluent enough to place focus naturally. The entire set of the sentences was repeated three times in a randomized order. Whenever a speaker misplaced focus or produced the intended IP (e.g., *‘Bed fast again’*) with a strong prosodic juncture inside, the speaker was asked to repeat the sentence a few more times to obtain a token with the best-matched intended focus or position. A total of 5184 tokens (36 speakers × 8 target words × 3 focus types × 2 positions × 3 repetitions) were obtained. The collected tokens were further checked by all three authors on the placement of pitch accent on the focused word, and position of the test word.^1^ Thirteen tokens were further discarded, as agreed by all three authors, due to inadequate prosodic junctures around the test word or a misplacement of pitch accent.

### Measurements

In order to investigate effects of focus and position on the acoustic realization of the English coda voicing contrast, four acoustic parameters were measured:

– *Duration of preceding vowel (V-duration)*: vowel duration was taken as the interval from the onset of the voicing of the preceding vowel (defined as the zero-crossing point before the first positive peak of the periodic waveform which is largely aligned with the onset of F1 seen in the spectrogram) to the vowel offset (defined as the point of abrupt discontinuity in the amplitude of the waveform which coincides with the offset of F2 seen in the spectrogram).
– *F1 and F2:* F1 and F2 of the vowels (/ε, æ/ as in *bed-bet* and *bad-bat*) were taken from the steady-state region (which was largely around the midpoint of the vowel) from the spectrographic displays.^2^ Although much discussion on the coda voicing effect on formants has been based on the results obtained with F1, we included both F1 and F2 as they both have been found to show coda voicing effects on English monophthongs (Wolf, 1978; Summers, 1988). The values were obtained by using an LPC formant tracking function with hand corrections based on visual inspection of the spectrogram for each token.
– *Voice Onset Time (VOT):* VOT of the voiceless (aspirated) stop /p/ in the syllable onset position (*ped-pet, pad-pat)* was taken from the point of the stop release to the voice onset of the vowel (as defined for the vowel duration measure). VOTs for voiced stops were not included partly because they could often be negative (voice lead) adding an additional complexity, and partly because in an ‘aspiration’ language like English the already short VOTs for voiced stops are not expected to vary much as a function of various factors (e.g., Kessinger and Blumstein, 1997; Smiljanić and Bradlow, 2008).

### Statistical Analyses

In order to evaluate statistically the effects of prosodic factors and vowel context on English coda voicing as produced by different groups of speakers, a series of repeated measures Analyses of Variance (RM ANOVA) was conducted using SPSS 21 statistical package for windows on the acoustic measures mentioned above. At first, statistical analyses were performed separately for each language group (Native American English, NAE, vs. Native Korean, NK) with four within-subject factors, Voicing (voiced /d/ vs. voiceless /t/ coda), Vowel type (V-type: /ε/ vs. /æ/), Focus (PH-FOC vs. LEX-FOC vs. NoFOC), and Position (IP-initial vs. IP-medial). For the NK group, there was a between-subject factor, Group (NK-advanced vs. NK-intermediate). Combined analyses were then conducted, with four within-subject factors listed above and one between-subject factor, Native Language (NAE vs. NK). When there were interactions between factors, *post hoc* pairwise comparisons were performed with Bonferroni/Dunn corrections. *p*-values less than 0.05 were considered statistically significant, and the values between 0.05 and 0.08 were treated as a trend. In the following section, we first outline the results, present the statistical results separately by each language group (NAE vs. NK) for each of the acoustic parameters, and provide combined analyses with both language groups.

## Results

### Vowel Duration

**FIGURE 1:**
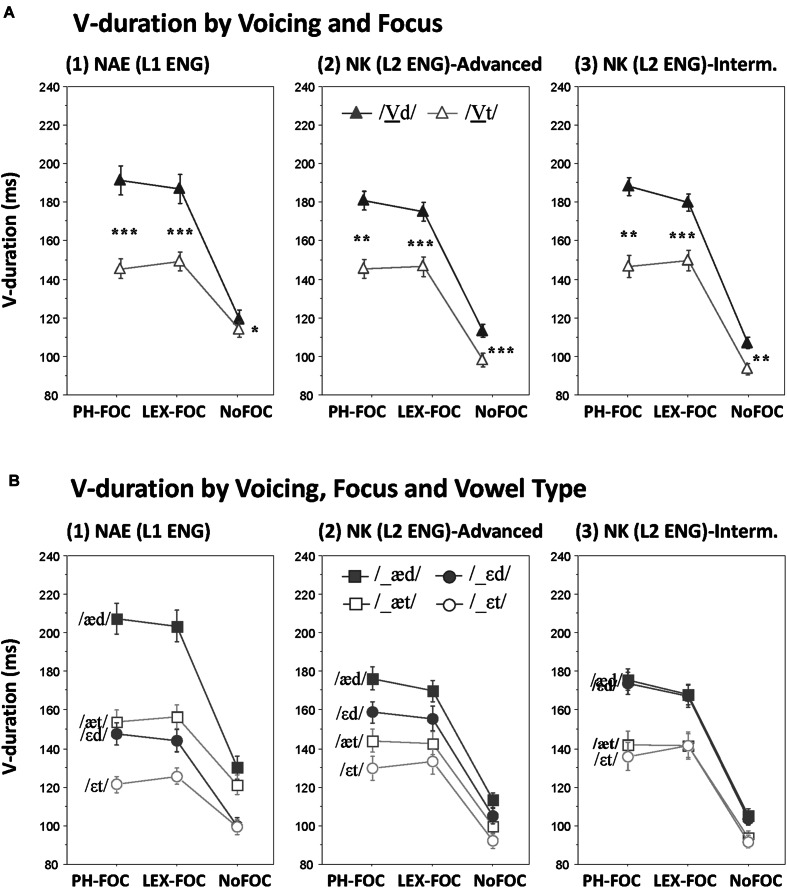
**Effects of coda voicing on vowel duration. (A)** Voicing × Focus interactions; **(B)** Voicing × Focus × Vowel type interactions, as produced by (1) native speakers of English, (2) Korean advanced learners of English, and (3) Korean intermediate learners of English (^∗∗∗^*p* < 0.001, ^∗∗^*p* < 0.01, ^∗^*p* < 0.05).

Effects of Voicing on V-duration and its possible interactions with Focus and Vowel Type are illustrated for each speaker group in **Figure 1**. As can be visually observed in **Figure 1A**, both the NAE (native American English) and the NK (native Korean) speakers showed robust coda voicing effects on V-duration. Crucially, the effect was augmented in the focused conditions but attenuated in the unfocused conditions across the board. The figure also shows the interaction between Voicing and Focus was further conditioned by the speakers’ native language: the focus-induced augmentation of the coda voicing effect on vowel duration tended to be greater for the NAE speakers (**Figure 1A1**) than for the NK speakers (**Figures 1A2,3**), whereas the reverse was true in the unfocused (NoFOC) condition in which the lengthening effect was more extremely attenuated by the NAE speakers than by the NK speakers (both advanced and intermediate). Furthermore, it is observable from **Figure 1B** that the NAE speakers maintained a clear durational division for the intrinsic vowel height between the mid and the low vowels (/ε/ vs. /æ/; **Figure 1B1**), but that the division was less clear for the NK-advanced speakers (**Figure 1B2**) and it entirely disappeared for the NK-intermediate speakers (**Figure 1B3**), while the difference in V-duration due to coda voicing remained unchanged. These observations were statistically supported by RM ANOVAs as reported below.

#### Effects on V-Duration by NAE (L1 ENG)

The NAE speakers showed a main effect of Voicing on V-duration, such that the vowel was longer before a voiced than before a voiceless stop (/d/ vs. /t/; mean difference 25.8 ms, *F*[1,11] = 57.4, *p* < 0.001). The Voicing effect, however, interacted with Focus (*F*[2,22] = 46.9, *p* < 0.001). As shown in **Figure 1A1**, the Voicing by Focus interaction stemmed from a focus-sensitive Voicing effect: the Voicing effect was augmented in the focused conditions [PH-FOC, mean difference 39.9 ms, *t*(11) = 59.2, *p* < 0.001; LEX-FOC, mean difference 32.8, *t*(11) = 56.9, *p* < 0.001] while the effect was extremely attenuated in the unfocused condition [NoFOC, mean difference 4.7 ms, *t*(11) = 5.9, *p <* 0.05]. Furthermore, the interaction appeared to be in part due to, on the average, a larger Voicing effect in the phonologically focused (PH-FOC) than in the lexically focused (LEX-FOC) condition (39.9 ms vs. 32.8 ms). There was also a three-way interaction between Voicing, Focus and Vowel Type (*F*[2,22] = 17.2, *p <* 0.001), such that the focus-sensitive voicing effect on V-duration was further conditioned by Vowel Type: as can be seen in **Figure 1B1**, there was a small but significant Voicing effect on V-duration for /æ/ in the NoFOC condition [NoFOC, mean difference 8.9 ms, *t*(11) = 3.3, *p* < 0.01] but not for /ε/ [NoFOC, mean difference 0.4 ms, *t*(11) = 0.3, *p* = 0.79]. Another noteworthy observation was that while the Voicing effect was robust for both vowel types (/ε/ vs. /æ/), there was a significant interaction between Voicing and Vowel Type (*F*[1,11] = 41.3, *p <* 0.001). As can be inferred from **Figure 1B1**, the interaction was due to the fact that the coda voicing effect was larger for the low vowel /æ/ [mean difference 36.5 ms, *t*(11) = 70.3, *p* < 0.001] than for the mid vowel /ε/ [mean difference 15.1 ms, *t*(11) = 23.2, *p* < 0.01], presumably because the intrinsically longer (low) vowel has a greater degree of freedom for temporal expansion. It is also worth mentioning that there was a four-way interaction which included the Position factor: Voicing × Focus × Vowel Type × Position (*F*[2,22] = 13.1, *p* < 0.01). The four-way interaction, however, was too complicated to be fully understood, but a visual inspection indicated that one of the contributing patterns (figure not shown) to the interaction was that the Voicing effect on the duration of /æ/ in the NoFOC condition turned out to have stemmed mostly from a robust voicing effect on /æ/ in the IP-initial position [/æ/, NoFOC, IP-initial, mean difference 11.7 ms, *t*(11) = 3.6, *p* < 0.01; NoFOC, IP-medial, mean difference 6.2 ms, *t*(11) = 1.8, *p* = 0.09].

#### Effects on V-Duration by NK (L2 ENG)

Native Korean speakers also showed a robust main effect of Voicing on V-duration, such that it was longer before a voiced than before a voiceless stop (mean difference 23.8 ms, *F*[1,22] = 53.43, *p* < 0.001). As was the case with the NAE speakers, the Voicing effect interacted with Focus (Voicing × Focus, *F*[1,44] = 16.1, *p* < 0.001) due to the fact that the Voicing effect was augmented in the focused conditions [PH-FOC, mean difference 33.4 ms, *t*(23) = 6.2, *p* < 0.001; LEX-FOC, mean difference 25.4 ms, *t*(23) = 7.3, *p* < 0.001], but attenuated in the unfocused (NoFOC) condition [mean difference 12.5 ms, *t*(23) = 7.2, *p* < 0.001]. Furthermore, as was the case with the NAE speakers, the NK speakers also showed a similar tendency toward a larger Voicing effect in the phonologically focused (PH-FOC) than in the lexically focused (LEX-FOC) conditions (33.4 ms vs. 25.4 ms). This interaction was observed for both NK-advanced and NK-intermediate speakers as visually shown in **Figures 1A2,3** and statistically confirmed—i.e., there was no further interaction with Group (Voicing × Focus × Group, *F*[2,44] < 1, *p* > 0.6).

There was no other interaction effect that involved Voicing, except for a Voicing × Position interaction (*F*[1,22] = 4.92, *p* < 0.05). Planned *t*-tests, however, indicated that there was no noticeable difference in the Voicing effect on V-duration as a function of Position [IP-initial, mean difference 22.4 ms, *t*(23) = 7.1, *p* < 0.001; IP-medial, mean difference 25.2 ms, *t*(23) = 7.5, *p* < 0.001]. This suggests that Position did not heavily modulate the temporal variation of the vowel due to coda voicing. It is also worth mentioning that there was a significant interaction between Vowel and Group: the NK-advanced speakers marked the intrinsic durational difference between /ε/ and /æ/ [mean difference 11.97, *t*(11) = 7.5, *p* < 0.05; see **Figure 1B2**] while the NK-intermediate speakers showed a complete overlap between the two vowels [mean difference 2 ms, *t*(11) = 1.74, *p* > 0.2; see **Figure 1B3**]. Thus, although the NK-intermediate speakers failed to use the vowel duration cue for the intrinsic vowel height difference, they used the cue successfully for marking the phonological voicing contrast of the following stops even in the non-prominent (unfocused) context.

#### Combined Analyses on V-Duration Across NAE (L1 ENG) and NK (L2 ENG)

The above-observed patterns on the coda voicing effects on V-duration were based on RM ANOVAs, separately carried out for the NAE and NK speakers. The results of a combined analysis (a five-way ANOVA) with an additional factor Native Language (NAE vs. NK) indeed showed a significant three-way interaction: Voicing × Focus × Language (*F*[2,68] = 4.197, *p <* 0.05). As seen in **Figure 1A**, the augmented Voicing effect on V-duration in the focused condition was on the average *larger* for the NAE than for the NK speakers (PH-FOC, 39.9 ms vs. 33.4 ms; LEX-FOC, 32.8 ms vs. 25.4 ms), whereas the attenuated voicing effect in the unfocused conditioned was on the average *smaller* for the NAE than for the NK speakers (NoFOC, 4.7 ms vs. 12.5 ms). The results of the combined analysis also showed a four-way interaction: Voicing × Focus × Vowel Type × Language (*F*[1,68] = 9.44, *p* < 0.001). As seen in **Figure 1B**, the NAE speakers showed a three-way interaction between Voicing, Focus and Vowel Type while the NK speakers did not.

### Voice Onset Time

**FIGURE 2:**
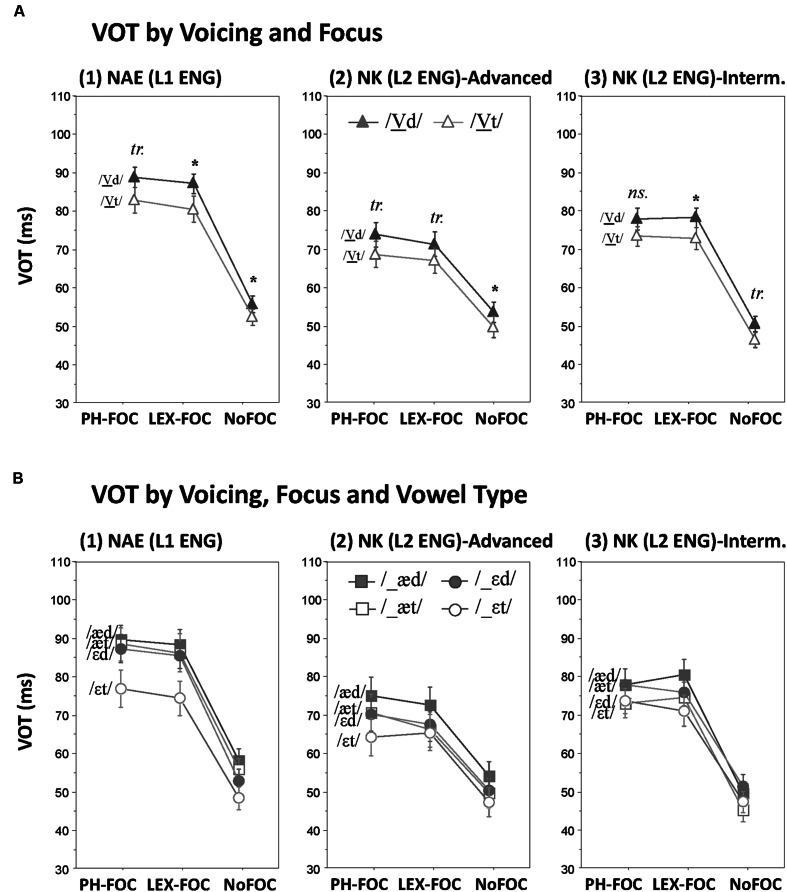
**Effects of coda voicing on VOT. (A)** Voicing × Focus interactions; **(B)** Voicing × Focus × Vowel type interactions, as produced by (1) native speakers of English, (2) Korean advanced learners of English, and (3) Korean intermediate learners of English (*tr.* = *p* < 0.08, ^∗^*p* < 0.05).

Effects of Voicing on VOT and its possible interactions with Focus and Vowel Type are illustrated for each speaker group in **Figure 2**. As can be visually observed in **Figure 2A**, the most striking pattern was that VOT (which may be taken as the initial component of the articulatory vocalic gesture in the temporal dimension) was indeed influenced by the voicing of the following coda, such that VOT for the voiceless stop (/p/) was on the average longer before a voiced than before a voiceless coda (/d/ vs. /t/) for both the NAE and the NK groups. Unlike the Voicing effect on V-duration, however, the results for both the NAE and the NK speakers did not show a noticeable interaction between Voicing and Focus. (Recall that the voicing effect on V-duration was augmented in the focused condition but attenuated in the unfocused condition). **Figure 2B** shows a possible difference that came from the speakers’ native languages, especially in terms of whether Voicing further interacted with Focus and Vowel Type. As can be seen in **Figure 2B1**, for the mid vowel /ε/ (but not for the low vowel /æ/), the NAE speakers indeed show an augmented Voicing effect on VOT in the *focused* conditions (PH-FOC and LEX-FOC) as compared with the Voicing effect in the *unfocused* (NoFOC) condition. The NK speakers, as can be seen in **Figures 2B2,3**, showed no such interaction, although the NK-advanced speakers shows some resemblance to the NAE’s interaction pattern. These observations were statistically supported by RM ANOVAs as reported below.

#### Effects on VOT by NAE (L1 ENG)

The NAE speakers showed a significant main effect of Voicing on VOT (*F*[1,11] = 9.7, *p <* 0.05) such that VOT for the voiceless stop /p/ in the onset position was longer when the coda was voiced than when it was voiceless (as shown in **Figure 2A1**). There was, however, a significant two-way interaction between Voicing and Vowel Type (*F*[1,11] = 8.3, *p* < 0.05). Planned *t*-tests indicated that the two-way interaction stemmed from the fact that the Voicing effect was reliable only for the mid vowel /ε/ [mean difference 8.7 ms, *t*(11) = 4.2, *p* < 0.01], but not for the low vowel /æ/ [mean difference 1.9 ms, *t*(11) = 0.9, *p* = 0.37], as can be seen in **Figure 2B1**. There was also a three-way interaction between Voicing, Focus and Vowel Type (*F*[2,22] = 3.8, *p* < 0.05) which was due to the fact that the Voicing effect on VOT before the mid vowel /ε/ was larger in the focused conditions than in the unfocused (NoFOC) conditions [PH-FOC, mean difference 10.4 ms, *t*(11) = 3.4, *p* < 0.01; LEX-FOC, mean difference 11.2, *t*(11) = 3.1, *p* < 0.05; NoFOC, mean difference 4.4 ms, *t*(11) = 2.6, *p* < 0.05]. There was no other significant interactions that involved the Voicing factor.

#### Effects on VOT by NK (L2 ENG)

Like the NAE speakers, the NK speakers showed a significant main effect of Voicing on VOT (*F*[1,22] = 20.64, *p* < 0.001), such that VOT for the voiceless stop in the onset position was longer when the coda was voiced than when it was voiceless (**Figures 2A2,3**). Unlike the case with the NAE speakers, the Voicing effect did not interact with Vowel Type and Focus: there was no Voicing by Vowel Type interaction (*F*[2,44] < 1, *p* > 0.3), nor was there a three-way interaction between Voicing, Focus and Vowel Type (*F*[2,44] < 1, *p* > 0.5), as can be inferred from **Figures 2B2,3**. There was no further interaction with Group, indicating that speakers of both the NK-advanced and the NK-intermediate groups did not modulate the Voicing effect as a function of Focus and Vowel Type. There was no other significant interaction that involved the Voicing factor.

#### Combined Analyses on VOT Across NAE (L1 ENG) and NK (L2 ENG)

A combined analysis with Native Language as an additional factor returned a significant main effect of Voicing (*F*[1,34] = 28.26, *p <* 0.001) with no interaction between Voicing and Language (*F*[1,64] < 1, *p* > 0.7). This confirmed the robust coda voicing effect on the onset VOT across speakers of both native and non-native groups (NAE and NK). The combined analysis also showed a significant three-way interaction: Voicing × Vowel Type × Language (*F*[1,34] = 11.7, *p* < 0.005), reflecting the fact that the NAE speakers showed an interaction between Voicing and Vowel (i.e., the voicing effect was significant only for the mid vowel /ε/), while the NK showed the effect for both /ε/ and /æ/. However, there was no four-way interaction of Voicing × Focus × Vowel Type × Language (*F*[2,68] = 1.83, *p* > 0.1), despite the fact that there was a significant three-way interaction of Voicing × Focus × Vowel Type for the NAE speakers, but not for the NK speakers. Thus, the results of the combined analysis indicated that the differential Voicing effects on VOT as a function of speakers’ native language was most reliably evident in the presence or absence of a Voicing × Vowel interaction for NAE vs. NK.

### F1

**FIGURE 3:**
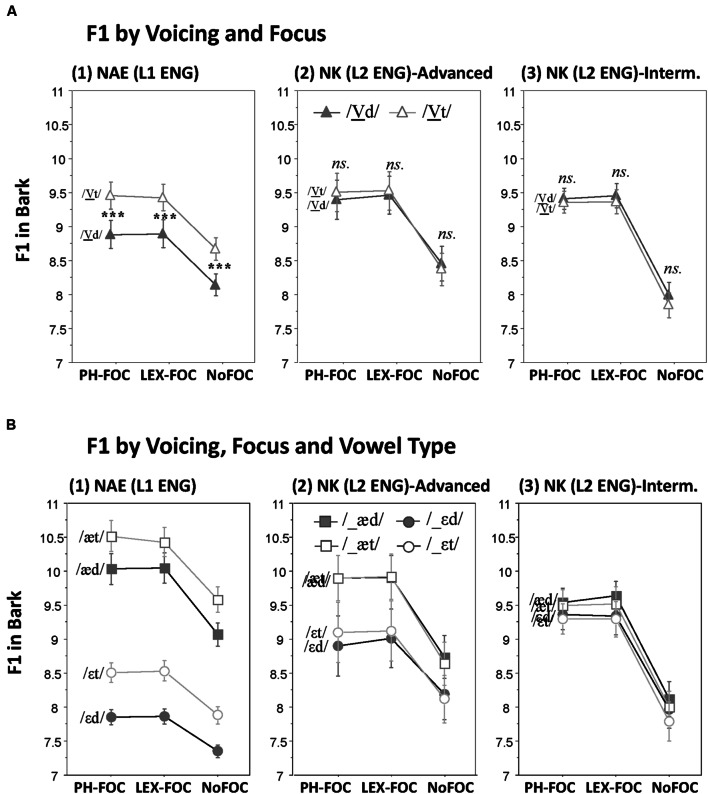
**Effects of coda voicing on F1. (A)** Voicing × Focus interactions; **(B)** Voicing × Focus × Vowel type interactions, as produced by (1) native speakers of English, (2) Korean advanced learners of English, and (3) Korean intermediate learners of English (^∗∗∗^*p* < 0.001).

Effects of Voicing on F1 and its possible interactions with Focus and Vowel Type are illustrated for each speaker group in **Figure 3**. As can be visually observed in **Figure 3B1**, the NAE speakers employed the F1 cue not only for marking the phonemic contrast between the mid vowel and the low vowel (/ε/-/æ/), but also for marking the voicing contrast of the following codas with F1 being lower before a voiced than before a voiceless coda, thus positioning the vowels higher in the vowel space. On the other hand, the NK speakers (**Figures 3A2,3**) did not use the F1 cue at all for marking the coda voicing contrast, although the NK-advanced speakers did use the F1 cue for making a distinction between the mid and the low vowels. These observations were statistically supported by RM ANOVAs as reported below.

#### Effects on F1 by NAE (L1 ENG)

There was a main effect of Voicing on F1 of the preceding vowel (*F*[1,11] = 70.9, *p* < 0.001), such that F1 was lower (thus positioning the vowel higher in the vowel space) before a voiced than before a voiceless coda (/d/ vs. /t/; see the general pattern in **Figure 3A1** for NAE). Unlike V-duration, F1 showed no Voicing by Focus interaction (*F*[2,22] < 1, *p* > 0.4), suggesting that the Voicing effect (lower F1 before a voiced coda) remained unchanged across different focus types (as can be seen in **Figure 3A1**). RM ANOVAs also returned a significant three-way interaction between Voicing, Focus and Vowel Type (*F*[2,22] = 3.6, *p* < 0.05), but planned *t*-tests indicated that the Voicing effect on F1 remained significant in each focus condition for each vowel type (all at *p* < 0.001) showing the same direction. As can be visually inferred from **Figure 3B1**, the three-way interaction effect appeared to have stemmed from the fact that the F1 difference due to coda voicing was on the average larger in the phonologically focused (PH-FOC) than in the lexically focused (LEX-FOC) for /æ/ (mean difference 0.49 vs. 0.38 Bark, respectively), but not for /ε/ (mean difference 0.66 vs. 0.67 Bark, respectively). (Compare the F1 difference in the PH-FOC vs. LEX-FOC conditions for the /εd/-/εt/ pair vs. the /æd/-/æt/pair in **Figure 3B1**).

#### Effects on F1 by NK (L2 ENG)

For NK speakers, there was no main effect of Voicing on F1 (*F*[1,22] < 1, *p* > 0.1) nor was there any interaction between factors that involved Voicing. In particular, the fact that the Voicing factor did not interact with Group (NK-advanced vs. NK-intermediate; *F*[1,22] = 1.87, *p* > 0.1) indicates that neither group of NK speakers employed the F1 cue for marking the coda voicing contrast. This null voicing effect on F1 is illustrated in **Figures 3A2,3** for each group. Furthermore, it is interesting to note that the NK-advanced speakers made a clear phonemic distinction between /ε/ and /æ/ (**Figure 3B2**), although the NK-intermediate speakers did not (**Figure 3B3**). But they both failed to use the F1 cue for the coda voicing contrast. In other words, the NK-advanced speakers did use the F1 cue for the phonemic vowel contrast, but not for the voicing contrast of the following codas. A visual inspection of the results, however, suggested that the NK-advanced speakers may possibly employ the F1 cue for the voicing contrast at least in one particular condition—i.e., in the PH-FOC condition for the /εd/-/εt/ pair, as can be seen in **Figure 3B2**. Planned *t*-tests indeed showed that there was a small but significant voicing effect only in this particular condition (*F*[1,11] = 5.0, *p* < 0.05).

#### Combined Analyses on F1 Across NAE (L1 ENG) and NK (L2 ENG)

As reported above, the results of RM ANOVAs run separately for each native language group (NAE and NK) showed clearly that the spectral F1 cue for the coda voicing contrast was employed by the NAE but not by the NK speakers. A five-way RM ANOVA with Native Language as an additional factor returned a significant Voicing and Language interaction on F1 (*F*[1,34] = 51.4, *p* < 0.001) confirming the speakers’ differential use of the F1 cue as a function of their native language.

### F2

**FIGURE 4:**
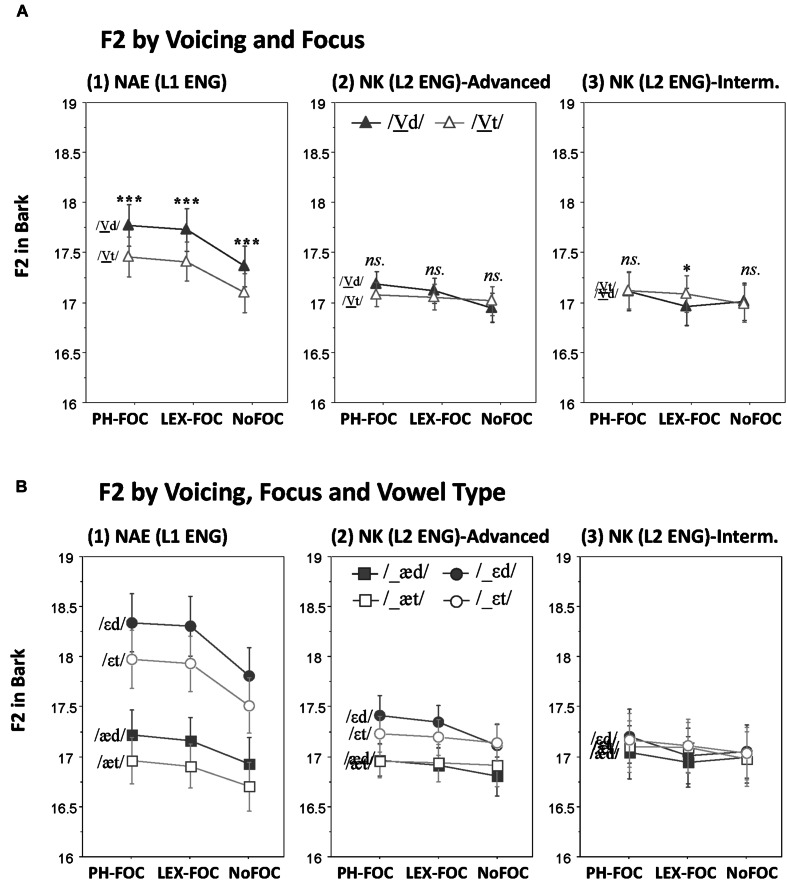
**Effects of coda voicing on F2. (A)** Voicing × Focus interactions; **(B)** Voicing × Focus × Vowel type interactions, as produced by (1) native speakers of English, (2) Korean advanced learners of English, and (3) Korean intermediate learners of English (^∗∗∗^*p* < 0.001, ^∗^*p* < 0.05).

Effects of Voicing on F2 and its possible interactions with Focus and Vowel Type are illustrated for each speaker group in **Figure 4**. As can be visually observed in the figure (and as was the case with F1), the NAE speakers employed the F2 cue for marking both the phonemic contrast (/ε/-/æ/; **Figure 4B1**) and the voicing contrast of the following codas with F2 being higher before a voiced coda (**Figure 4A1**), thus positioning the vowels more advanced in the vowel space before a voiced than before a voiceless coda. On the other hand, the NK speakers did not use the F2 cue at all for marking the coda voicing contrast (**Figures 4A2,3**), although the NK-advanced speakers did use the F2 cue for making a distinction between the mid and the low vowels (/ε/ vs. /æ/; **Figure 4B2**). These observations were statistically supported by RM ANOVAs as reported below.

#### Effects on Voicing by NAE (L1 ENG)

The NAE speakers showed a main effect of Voicing on F2, such that F2 was higher before a voiced coda /d/ than before a voiceless coda /t/ (*F*[1,11] = 40.4, *p* < 0.001), which positioned the vowel before a voiced coda more advanced in the vowel space (**Figure 4A1**). As was the case with F1, F2 also showed a vowel-independent voicing effect. That is, there was no interaction between Voicing and Vowel Type (*F*[1,11] = 2.55, *p* > 0.1), indicating that the Voicing effect on F2 (higher F2 for a voiced coda) was applicable to both the mid vowel /ε/ and the low vowel /æ/ as shown in **Figure 4B1**. Just like on F1, the Voicing effect on F2 did not interact with Focus (*F*[2,22] < 1, *p* > 0.9), nor was there a three-way interaction between Voicing, Focus and Vowel Type (*F*[2,22] < 1, *p* > 0.8), indicating no further modulation of the Voicing effect as a function of Focus and Vowel Type. Voicing did not interact with Position, either (*F*[1,11] < 1, *p* > 0.9), showing a position-independent voicing effect (figure not shown). There was no other interaction effect that involved Voicing.

#### Effects on Voicing by NK (L2 ENG)

Unlike the NAE speakers, the NK speakers did not generally employ the F2 cue in marking the coda voicing contrast: there was no main effect of Voicing on F2 (*F*[1,22] < 1, *p* > 0.9). There was no significant interaction between Voicing and Group (*F*[1,22] < 1, *p* > 0.3), either, indicating that both the NK-advanced and NK-intermediate speakers failed to use the F2 cue. There was, however, a three-way interaction: Voicing × Focus × Group (*F*[2,44] = 3.27, *p* < 0.05). The interaction was due to the fact that while the NK-advanced speakers showed no Voicing effect on F2 in each focus condition (**Figure 4A2**), the NK-intermediate speakers showed a significant Voicing effect in the LEX-FOC condition (**Figure 4A3**). But as can be seen in **Figure 4A3**, the Voicing effect in the LEX-FOC condition was the opposite of what was found for the NAE speakers. It is also interesting to note that just as they used F1, the NK-advanced speakers also used F2 to make a phonemic distinction between /ε/ and /æ/ (*F*[1,11] = 6.0, *p* < 0.05; **Figure 4B2**), resembling the NAE’s /ε/-/æ/ distinction, whereas the NK-intermediate speakers did not (*F*[1,11] = 1.04, *p* > 0.3). The NK-advanced speakers, however, failed to utilize the F2 cue for the coda voicing contrast. There was no other interaction that involved the Voicing factor.

#### Combined Analyses on F2 Across NAE (L1 ENG) and NK (L2 ENG)

As reported above, the results of RM ANOVAs run separately for each native language group (NAE and NK) showed clearly that the spectral F2 cue for the coda voicing contrast was employed by the NAE but not by the NK speakers. A five-way RM ANOVA with Native Language as an additional factor returned a significant Voicing and Language interaction on F2 (*F*[1,34] = 25.46, *p* < 0.001) confirming the speakers’ differential use of the F2 cue as a function of their native language.

## Discussion

In the present study we investigated how coda voicing contrast in English would be manifested in the acoustic-phonetic detail of the preceding vowel in both the temporal and the spectral dimensions. Crucially, we compared speech productions in L1 (by 12 native speakers of American English, NAE) and L2 (by 24 non-native Korean learners of English, NK) with a view to understanding the phonetics-prosody interface in L1 and L2. To this end, we tested effects of prominence that stemmed from prosodic structure closely related to information structure as reflected in different focus types: phonological focus (PH-FOX), lexical focus (LEX-FOC), and no focus (NoFOC). We also controlled for the prosodic position factor, so that the test words in different focus conditions occurred both in the phrase-initial and the phrase-medial positions (i.e., the IP-initial vs. the IP-medial position). In what follows, we recapitulate several important findings that have emerged from the results along with some discussion on implications for phonetic encoding of phonological contrast and its interaction with higher order linguistic structure in L2 speech.

### Differential Use of Phonetic Dimensions in L1 vs. L2

One of the basic findings of the present study is that both the native (NAE) and the non-native (NK) speakers showed robust coda voicing effects on the temporal realization of the preceding vowel. The vowel duration was systematically longer before a voiced than before a voiceless coda stop in the production of both the NAE and the NK speakers. The effect was independent of the prosodic position (IP-initial vs. IP-medial) in which the target bearing word occurred. (See the next section for discussion on an interaction of voicing and focus.) The coda voicing effect in the temporal dimension was further evident in the syllable-onset VOT. Both the native (NAE) and the non-native (NK) speakers showed a significant main effect of coda voicing on the syllable-onset VOT which was longer before a voiced than before a voiceless coda. Interestingly, however, the NAE speakers showed an interaction effect on VOT between Voicing and Vowel: the NAE speakers showed the voicing effect on VOT for the mid vowel pair (*ped-pet*), but not for the low vowel pair (*pad-pat*), whereas the non-native (NK) speakers showed no such interaction. We do not have any principled explanation to offer for why there is such an asymmetric coda voicing effect on VOT in the native (NAE) speakers’ production, but one cannot entirely rule out the possibility that the asymmetry has stemmed from the lexical differences (e.g., word frequency) between the two pairs. On the other hand, the fact that the non-native (NK) speakers showed a consistent coda voicing effect on VOT regardless of the word pair may then be interpretable as stemming from the possibility that non-native speakers are less sensitive to the lexical differences in speech production. While these possibilities need further corroborations, what appears to be clear is that the coda voicing effect on the syllable-onset VOT is less robust than that on the vowel next to the coda at least for the NAE speakers, possibly reflecting the proximity effect—i.e., VOT is not adjacent to the source of coda voicing.

From an articulatory gestural point of view, as discussed in the introduction, VOT may be taken to be part of the vowel, given that the onset of the vocalic opening gesture for the vowel coincides with the onset of VOT. The lengthened VOT before a voiced coda therefore suggests that coda voicing affects the entire temporal structure of the vowel (cf. Pycha and Dahan, 2016), rather than being localized to a later part of the vowel (cf. Chen, 1970). From an acoustic point of view, on the other hand, VOT is considered as part of the syllable-onset voiceless stop, so that the effect on VOT defined as such further implies that coda voicing may modify the temporal structure of the entire syllable even beyond the preceding vowel. This rather long distant effect is in line with the case for the syllable-onset /l/ whose phonetic realization was found to be modulated by the voicing of the syllable-coda (e.g., Nguyen and Hawkins, 2004). Importantly, although the non-native (NK) speakers have no experience with such a phonological coda voicing contrast in their native (Korea) language, they appear to modulate the temporal structure of the entire vowel (or possibly the entire syllable) in a comparable way as the native speakers do.

Unlike the coda voicing effects in the temporal dimension, however, the way that coda voicing contrast was manifested in the spectral dimension was clearly bifurcated between L1 (NAE) and L2 (NK) speaker groups. The native (NAE) speakers showed robust effects of coda voicing on both F1 and F2 for the monophthong vowels /ε, æ/ largely in line with the previous studies (Wolf, 1978; Summers, 1987; Crowther and Mann, 1992). Both the mid and the low vowels /ε, æ/ were produced with *lower* F1 and *higher* F2 before a voiced than a voiceless stop (thus positioning the vowel *higher* (lower F1) and *more advanced* (higher F2) before a voiced stop in the acoustic vowel space). It is also worth pointing out that previous studies observed lower F1 before a voiced coda only for low vowels (/æ/ or /ɑ/) with an interpretation that the coda voicing effect was due to ‘hyperarticulation’ of the vowel before a *voiceless* coda (as reflected in *higher* F1 before a voiceless coda and *lower* F1 before a voiced one), possibly enhancing the [+low] feature for the low vowel (e.g., see Thomas, 2000; Moreton, 2004 for a related discussion). The present study demonstrated that the same holds for the non-low (mid) vowel /ε/, indicating that the assumed hyperarticulation does not necessarily enhance the vowel’s distinctive feature—i.e., the increase in F1 before a voiceless stop is taken to enhance the [+low] for the low vowel /æ/, but not for the mid vowel /ε/.

Most crucially, however, unlike the NAE speakers, the non-native (NK) speakers did not show any evidence of their use of spectral cues to the coda voicing contrast. A question that arises here is then why there is discrepancy in the way that the non-native (NK) speakers employ the temporal dimension vs. the spectral dimension for encoding coda voicing contrast in L2 English—i.e., they successfully encode coda voicing contrast in the temporal dimension, but fail to do so in the spectral dimension. The asymmetric use of the temporal vs. the spectral dimension by the non-native (NK) speakers may be accounted for by different natures of the phonetic cues in the temporal vs. the spectral dimensions. On the one hand, as Bohn (1995) noted, cues in the temporal dimension may be taken to be perceptually more salient. The temporal dimension in fact is exploited to express a wide range of linguistic contrast (whether syntagmatic or paradigmatic) across languages (see Cho, 2015 for a review), presumably because of its universally driven perceptual salience. This view is consistent with previous observations: speakers rely more on temporal cues in processing an unfamiliar language (e.g., Tyler and Cutler, 2009; Kim et al., 2012); and infants are indeed sensitive to prosodic variation of speech input (including variation along the temporal dimension) even at an embryonic stage of L1 acquisition, and exploit prosodic cues in lexical segmentations (see Johnson, 2016 for a review). Furthermore, the fact that the coda voicing effect on the preceding vowel duration is a near-universal tendency (e.g, Chen, 1970; Keating, 1985; Maddieson, 1997) implies that the temporal cue for the coda voicing in L2 is likely to be unmarked and hence easily accessible to non-native speakers. Thus, the universally applicable use of temporal dimension appears to make it easier for the non-native (NK) speakers to encode the coda voicing contrast along the temporal dimension.

The failure of using the spectral dimension, on the other hand, appears to have stemmed from the speakers’ native language experience. Specifically, this possibility is in line with the view that speakers of a language with a sparsely populated vowel space has larger Difference Limens (DLs, or Just Noticeable Differences), thus being less sensitive to a small change in formant frequencies than speakers of a language with a densely populated vowel space (see Kent and Read, 2002 for a related discussion). The observed null effect of coda voicing on F1 and F2 for the non-native (NK) speakers can therefore be interpreted as having stemmed from the NK speakers’ native language experience whose smaller vowel inventory induces perceptual insensitivity to formant frequencies. On a related point, it is also worth pointing out that the NK-advanced learners of English (but not the NK-intermediate speakers) indeed used the spectral cues (F1, F2) to make a categorical phonemic distinction between the mid and the low vowels /ε, æ/, though the phonetic distance between the two vowels was not as large as that produced by the native (NAE) speakers. But even the NK-advanced speakers failed to use the spectral cues in a finer-grained way for marking coda voicing contrast. This is again in line with the prediction regarding differential perceptual sensitivities as a function of the size of the vowel inventory of the speakers’ native language.

These possibilities, taken together, suggest that the difference in how NK speakers use temporal and spectral dimensions stems from the fact that, in this case, one of the cues is universally driven and the other is L1-specific. It is therefore plausible that phonetic encoding of phonological contrast in L2 is constrained by an intricate relationship between the universal applicability of a phonetic cue for a given contrast and the non-native speakers’ language experience.^3^

### The Phonetics-Prosody Interface with Reference to Information Structure in L1 vs. L2

Another important finding of the present study was that both the NAE and the NK speakers showed a significant Voicing × Focus interaction in the temporal dimension. The coda voicing contrast was temporally enhanced under prominence, such that the vowel lengthening effect due to coda voicing was augmented in the focused conditions (both phonologically focused and lexically focused) whereas the effect was extremely attenuated in the unfocused condition. In other words, insofar as the temporal dimension was concerned, both the native (NAE) and the non-native (NK) speakers showed a comparable phonetics-prosody interface as reflected in the interplay between the phonetic realization of the coda voicing contrast and the prosodic prominence factor. The way that coda voicing interacted with focus may be interpreted as being driven by an interaction of two important principles of the linguistic communicative system: *contrast maximization* and *effort minimization* (e.g., Lindblom, 1990; de Jong, 1995; Flemming, 1995). In the focused condition—i.e., when signaled by the prominence (accentuation) factor of the prosodic structure in connection with information structure, both the NAE and the non-native (NK) speakers *hyperarticulate* by making effort to maximize the distinctiveness of coda voicing contrast. In the unfocused condition—i.e., when the prosodic structure signals that voicing contrast is no longer the locus of information, they ease articulatory effort or *hypoarticulate*. Furthermore, the fact that both the NK-advanced and the NK-intermediate speakers showed a similar interaction pattern as the native (NAE) speakers did suggests that the interplay between phonetics and prosody in L2 speech operates in a communicatively optimized way, regardless of the non-native speakers’ English proficiency, by making reference to higher-order information structure.

Another noteworthy finding was that both the NAE and the non-native (NK) speakers showed a trend toward a greater enhancement of coda voicing contrast in the phonologically focused (PH-FOC) than in the lexically focused (LEX-FOC) condition consistent with findings of previous studies (e.g., de Jong and Zawaydeh, 2002; de Jong, 2004). This result has some implications for the interaction between information structure and prosodic structure. Even if the focus realization from information structure was mediated by a nuclear pitch accent as part of the prominence system in the prosodic structure, the same nuclear pitch accent induced a finer-grained phonetic effect as a function of focus type (see Mücke and Grice, 2014, for a related discussion). This suggests that the prosodic structure effect is fine-tuned by making reference to information structure. Furthermore, the fact that the non-native (NK) speakers show a similar pattern indicates that such a fine-tuning according to information structure is characteristic of a human linguistic system, and thus is readily reflected in L2 speech.

The interaction of coda voicing and focus on vowel duration, however, was further modulated by the speakers’ native language. The non-native (NK) speakers enhanced the coda voicing contrast in the focused conditions but not as much as the native (NAE) speakers did, and they reduced the coda voicing contrast in the unfocused condition but not as extremely as the native (NAE) speakers did. Recall that the NAE speakers, when in the unfocused condition, did not even show any vowel lengthening effect due to coda voicing for the mid vowel /ε/, while the non-native (NK) speakers consistently maintained the voicing contrast for the vowel in the unfocused condition. These results therefore suggest that the native (NAE) speakers use the acoustic temporal space along a hypo- to hyper-articulation continuum in a polarized way for optimization of communication efficacy, while the non-native (NK) speakers do not seem to utilize the space as efficiently as the native speakers do. In other words, although the non-native (NK) speakers do encode coda voicing contrast by making reference to information structure mediated by the phonetics-prosody interface, it appears that the native-like encoding of coda voicing requires a further phonetic fine-tuning of vowel duration in response to communicative functional load that stems from information structure.

The difference in the voicing by focus interaction between the native (NAE) and the non-native (NK) speakers, however, does not seem to be entirely attributable to the non-native speakers’ less efficient way of utilizing the phonetic space, but it may also be at least in part due to the constraint from the L2 system in which the way that the non-native (NK) speakers maintain the phonological voicing contrast is different from that of the native (NAE) speakers. In the present study, the native (NAE) speakers did not show an interaction between coda voicing and focus in the spectral dimension (F1 and F2), but they used the F1 and F2 spectral cues consistently, which helps preserving coda voicing contrast even in the unfocused condition. Thus, even an extreme reduction of the voicing effect in the temporal dimension in the L1 system (as was the case for /ε/ in the unfocused condition) is not detrimental to the maintenance of the phonological voicing contrast as the difference due to coda voicing is invariantly present in the spectral dimension of the speech signal. On the other hand, the non-native (NK) speakers did not employ the spectral cue to the coda voicing in their L2 system. With the lack of the spectral cue, too extreme a reduction of the voicing effect in the temporal dimension would undermine the phonological coda voicing contrast. In other words, an optimization of temporal realization of coda voicing in response to information structure appears to be constrained by the way that coda voicing is phonetically encoded in the L2 phonetic system, such that the phonological contrast is invariantly maintained. The phonetic optimization of the phonetics-prosody interface in the L2 phonetic system can therefore be taken to be modulated by the non-native speakers’ native language experience.

### Implications for Phonological Abstraction and Lexical Representation in L2 System

The fact that the NK speakers’ sensitivity to the spectral vs. the temporal dimension in L2 is modulated by L1 (Korean) sound system indicates that phonetic encoding of coda voicing contrast is internalized in their L2 system in an L1-specific way, so that NK speakers’ phonetic manifestations of phonological abstraction deviate from those of the native (NAE) speakers. Such an L1-specific abstraction appears to be further supported by our anecdotal observation that while the native (NAE) speakers showed an asymmetric coda voicing effect on VOT for the *pad-pat* vs. the *ped-pet* pair (presumably in part due to the lexical differences), non-native (NK) speakers showed a consistent effect on VOT regardless of lexical pair. Non-native (NK) speakers’ impoverished lexical knowledge therefore appears to increase the role of phonological abstraction in phonetic encoding, hence the across-the-board voicing effect, although this possibility is subject to corroboration by further studies.

These observations have some implications for the nature of lexicon in L2. Recent years have witnessed a constructive debate in the literature on the nature of lexical representations, especially regarding how much phonetic detail is stored in the lexicon. One of recent approaches to this question is an exemplar-based approach (e.g., Goldinger, 1996, 1997; Pierrehumbert, 2001, 2003). It generally assumes a phonetically rich lexicon which stores phonetically detailed exemplars of specific speech events (also known as ‘episodes’) in a multidimensional phonetic space. A phonological contrast then emerges as phonetic categories are formed as a result of generalizations over a frequency weighted distribution of exemplars. Such a model is especially useful in accounting for effects of lexical frequency and individual differences which are prevalent in both speech production and perception. The perception-based exemplars are used in speech production, so that a phonological contrast is phonetically encoded based on random sampling from the frequency weighted distribution of exemplars associated with different (contrastive) phonetic categories. Although the theory has been developed primarily based on L1 speech, phonetic categories in L2, in principle, should be formed in a similar way. To the extent that the theory holds, however, the results of the present study indicate that phonetic detail of exemplars stored in the lexicon in L2 (developed by NK speakers) is different from that in L1 lexicon. In other words, the phonetic dimensions along which the perceived exemplars form a category appear to be constrained by the L2 speakers’ native language experience—i.e., L2 speakers’ perceptual bias due to their L1 experience constrains the distribution of exemplars in a multidimensional phonetic space. Furthermore, the fact that NK speakers showed no clear English proficiency effect (and no lexical item effect on VOT) implies some degree of phonological abstraction, leading to a question as to the extent to which L2 speakers’ phonetic encoding is indeed based on random sampling from a frequency weighted distribution of exemplars from the phonetically rich lexicon.

## Conclusion

In the present study, we have demonstrated that the low-level phonetic encoding of phonological coda voicing contrast in L1 vs. L2 English (by Korean learners of English) is modulated by the prominence factor of prosodic structure in connection with information structure. Specifically, the results suggest that the non-native (NK) speakers’ phonetic encoding of coda voicing contrast is modulated by an intricate interaction between the universal-applicability of phonetic cues used for the contrast along the temporal dimension and the non-native speakers’ native language experience which constrains a finer-grained use of the spectral cue (presumably due to its scarcely populated vowel inventory). Furthermore, just like the native (NAE) speakers, the non-native (NK) speakers showed that their phonetic encoding of coda voicing was modulated by information structure mediated by the phonetics-prosody interface. Regardless of the non-native speakers’ English proficiency, the L2 use of the acoustic phonetic space was polarized in a communicatively efficient way, in response to functional loads dictated by information structure. This suggests that once the relative acoustic phonetic cue is learned, the phonetics-prosody interface by making reference to higher order information structure appears to follow relatively easily, presumably because such an interaction is characteristic of the human linguistic communicative system, not specific to an individual language. However, the communicative efficacy of using the temporal dimension in L2 by the non-native (NK) speakers appeared to be less optimal compared to that in L1 speech. We proposed that such difference is also attributable to the non-native (NK) speakers’ native language experience. Given that the spectral dimension is not used by the non-native (NK) speakers for marking coda voicing contrast, the communicative efficacy along the temporal dimension is achieved in a way that is not detrimental to the maintenance of the phonological contrast of the coda voicing. The non-native use of the temporal dimension therefore appears to be ‘optimized’ for a particular L2 communicative system by the NK speakers.

All in all, the present study has built on a gradually growing body of L2 phonetic literature with respect to the phonetics-prosody interface in L2. There is no doubt that speech production of L1 and L2 alike is modulated by a human communicative system in such a way that the information that comes from higher-order linguistic or information structure is encoded in speech signal, and it is eventually available to the listener. Nevertheless, our understanding of L2 speech has been fairly limited, especially with respect to how low-level phonetic realization is systematically modulated by higher-order linguistic structure. The results of the present study therefore have further implications for theories of L2 speech (e.g., Flege, 2003; Best and Tyler, 2007; Davidson, 2011), for which there appears to be much room for further development regarding how the low-level phonetic implementation interacts with prosodic structure, how higher level linguistic information is further mediated by the phonetics-prosody interface, and how such interactions are constrained by the L2 speakers’ native language experience.

## Author Contributions

JC: the first author, designed the study from the beginning, carried out the experiment, analyzed the data, and wrote up an earlier version of this manuscript; SK: the second author, participated in the study at every stage, designing the study, analyzing the data, interpreting the results, discussing the results, and editing earlier versions of the manuscript; TC: the corresponding author, supervised the entire project at every stage, and edited the entire manuscript with elaborations on introduction, research questions, predictions, results section, interpretations of the results, and discussion with implications.

## Conflict of Interest Statement

The authors declare that the research was conducted in the absence of any commercial or financial relationships that could be construed as a potential conflict of interest.

## Footnotes

1 The prosodic transcriptions employed here were based on the conventions of the English ToBI (tones and break indices; Beckman and Ayers, 1994, Unpublished; Beckman et al., 2005). In ToBI, a tone with “^∗^” or a starred tone (e.g., H^∗^ or L+H^∗^) refers to a pitch accent that falls on a lexically stressed syllable along with a higher-level (phrasal) stress. H^∗^ means that a tone rises and reaches its peak largely in the vowel without a noticeable low tone that precedes it whereas L+H^∗^ means that the starred high tone (H^∗^) is realized primarily on a lexically stressed syllable preceded by a low tone. In the present study, the pitch accent type observed with the target words was either H^∗^ or L+H^∗^.

2 Although effects on F1 and F2 could be more robust near the coda consonant, formant values at the edge of the vowel appeared to be quite variable due to V-to-C formant transitions. Furthermore, previous researchers (e.g., Wolf, 1978; Summers, 1988) indicated that the coda voicing effect on formants was reliably observed in steady-state parts of the vowel. Our initial informal inspection of formant values with some speakers’ tokens also indicated that the coda effects were robust even in the middle of the vowel. We therefore decided to include F1 and F2 measures at the midpoint (steady-state) of the vowel.

3 One might wonder whether it is the temporal difference due to coda voicing that is taught explicitly in EFL class in Korea, which, if so, would have influenced the NK-speakers’ performance. To our best knowledge, however, the vowel length difference due to coda voicing is never taught explicitly in both the primary and the secondary school.
